# The Irreversible Neurogenic Stress Cardiomyopathy During Large Supratentorial Brain Tumor Resection

**DOI:** 10.1007/s12028-019-00847-9

**Published:** 2019-10-09

**Authors:** Ewa Lechowicz-Glogowska, Agata Rojek, Wieslawa Duszynska

**Affiliations:** 1 Department of Anaesthesiology and Intensive Therapy, Section on Neurocritical Care, Wroclaw Medical University, Wrocław, Poland; 2 Department of Neurology, Program in Neurology, Wroclaw Medical University, Wrocław, Poland

## Case Presentation

A 63-year-old woman was qualified for an urgent, large supratentorial metastatic tumor of breast cancer resection. The patient’s initial Glasgow Coma Scale (GCS) was 14 points. Other comorbidities were as follows: ca mammae, hypertension and hypothyreosis. Diabetes and acute coronary syndrome (ACS) have never been diagnosed. According to the patient’s history, she did not suffer from pain in thorax and she was able to climb to the second floor without fatigue. Preoperative electrocardiographic (EKG) test was within a normal range, and due to the lack of any cardiac risk factors, echocardiography and stress test were not done preoperatively as a part of the routine testing. The patient received steroids preoperatively, as well as one dose at the time of surgery. Induction of anesthesia was started after the implementation of monitoring the heart rate (HR)-EKG (5 leads), invasive blood pressure measurement, saturation of the blood (SaO2)-pulsoxymetry, CO2 in the expiratory air (ETCO2),—capnography, bispectral index (BIS), train of four-myorelaxation monitoring, central body temperature. A central line catheter was inserted into the right jugular vein after patient’s intubation. The patient’s initial blood gases showed no abnormalities (SaO2-99%). After the intravenous (IV) induction of anesthesia (propofol, fentanyl and rocuronium), sevoflurane (minimal alveolar concentration 0.7–0.9), and an adjuvant dose of ketamine given by an IV infusion (0.2–0.3 mg/kg body weight/hour) was started.

BIS was maintained at a level of 40–50%. Normal body temperature, blood gases and electrolytes (Na, K, Mg) were maintained. Due to a slight drop in blood pressure, following induction of anesthesia, small doses of norepinephrine (NE) were infused (0.02–0.03 mcg/kg/min) to keep proper mean arterial pressure and intracranial perfusion pressure. No severe intra-operative bleeding was observed. Euvolemia was maintained. Normal heart function and HR of 70–80/min and systolic blood pressure (SBP) around 100 mmHg were noted till the 4th hour of the surgery.

**Fig. 1:**
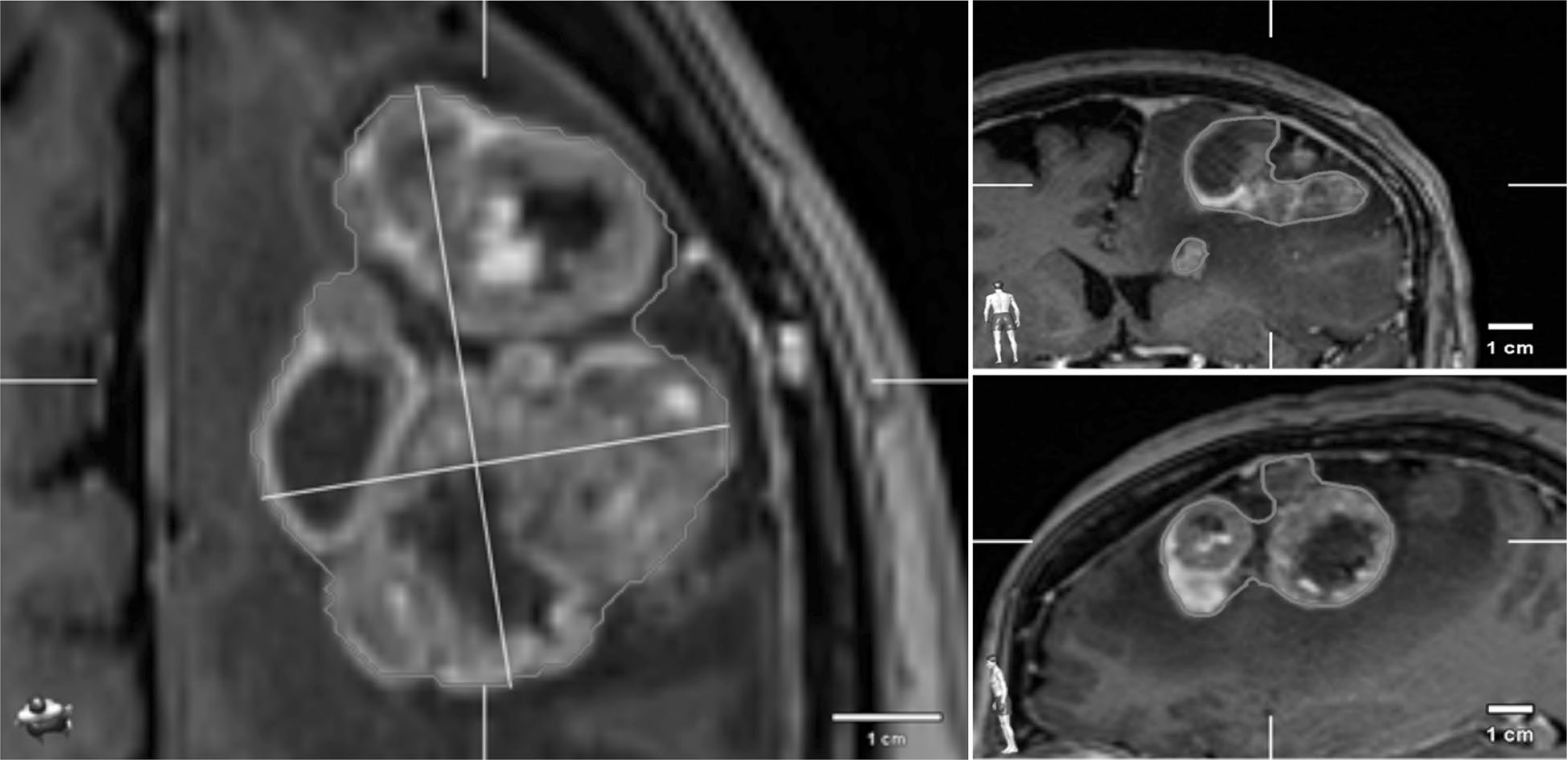
It shows an MRI scan of brain and solid tumor. Contrast was used during MRI examination

A sudden and significant HR increase up to 130/min, followed by a SBP drop to 80 mm Hg, appeared during the final surgery stage resection of the deepest tumor mass. Hyperglycemia (480 mg%) with severe metabolic acidosis (pH 7.19, lactate 4.9 mmol/L), oliguria and electrolyte abnormalities developed within minutes. Table 1 presents the patient’s biochemical parameters before, during and 24–48 h after the surgical procedure. NE (0.05–0.9 mcg/kg/min) with amiodarone was administered in a continuous IV infusion, followed by additional epinephrine (ADR) (0.18–1 mcg/kg/min), bicarbonates, insulin and electrolytes that were given in increasing doses. No stabilization was achieved in 2 h until the discontinuation of the tumor resection. Figure 1 shows the magnetic resonance imaging (MRI) scans of the brain and solid tumor using a contrast. This surgical procedure was carried out with neuro-navigation.

**Table 1:** Patient’s biochemical parameters before, during and 24, 48 h after surgery

Parameters	Before surgery	During surgery	24 h after surgery	48 h after surgery
Troponine I, pg/mL	–	64.9; 283.7	11,754.4	5433.7
BNP, pg/mL	–	–	2324.8	–
White blood cells, *n* × 10^3^/mm^3^	10.62	27.4	27.87	27.78
Platelet count, *n* × 10^3^/mm^3^	196	133	40	94
Glucose, mg/dL	142	375	142	258
Hb, g/dL	13.4	11.1	7.2	8.8
Ht, %	39.7	33.4	22.2	26.1
Serum creatinine, mg/dL	0.54	1.32	2.79	3.84
C-reactive protein, mg/L	0.64	–	19.27	–
Procalcitonin, ng/mL	–	–	21.04	24.1
Na, mEq/L	138	153	152	144
K, mEq/L	4.37	4.13	5.13	4.99
Prothrombin,%	112.2	74.5	27.8	48.38
PTT, s	19.4	22.09	34.35	36.73
Lactate, mmol/L		16	10.4	5.4
PaO_2_/FiO_2_		200	150	152

*BNP* blood natriuretic protein, *Hb* haemoglobin, *Ht* haematocrit, *PaO*_*2*_*/FiO*_*2*_ respiratory quotient, *PTT* partial thrombin time

The three metastatic tumors were close to each other and formed an expansive intracranial mass. This enabled the neurosurgeon to perform one-step resection surgery. The tumor removal was performed with no local complications such as increased bleeding or brain edema. The hemostasis was proper. Also, the hemostasis at the time of completion of the surgery was good as well as brain swelling was not observed.

As the surgical procedure was over, the patient was transferred to the intensive care unit (ICU) for further patient condition evaluation and treatment. After vasopressin (VP) (0.03 units/min) was added to the therapy (in the first hour of ICU stay), slow reduction of NE was possible. In the following hours, short circulatory stabilization was observed, ADR IV infusion was stopped, dobutamine (DBT) (5–10 mcg/kg/min) was added, and NE (0.3–0.7 mcg/kg/min) was continued. Cathecholamin and fluid therapy were used under the control of hemodynamic parameters, using transpulmonary thermodilution methods—pulse continuous cardiac output (PiCCO; PULSION medical System AG, Munich, Germany).

**Fig. 2:**
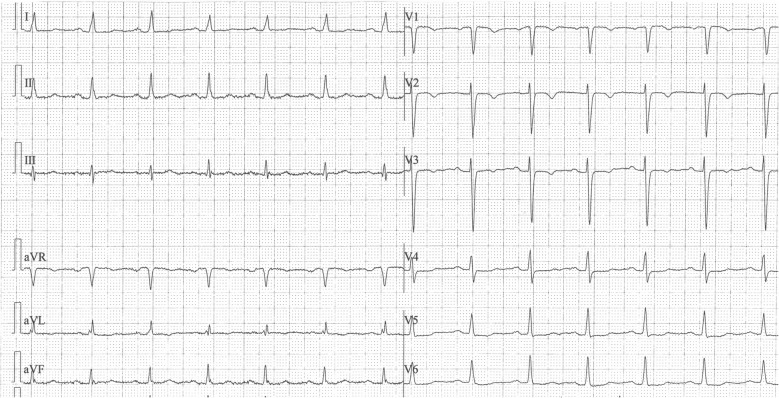
It shows EKG abnormalities: T waves inversion in leads V1–V3, flat T wave in the leads V4–V6 and I, aVL

Specific to cardiac injury, troponin (Troponin 1) was significantly increased (5433.7 pg/mL) as well as blood natriuretic protein (BNP) 2324.8 pg/mL. A postoperative EKG presented the new evolutions of lateral heart wall—T wave’s inversion in leads V1–V3, flat T wave in the leads V4–V6 and I, aVL (Fig. 2). The transthoracic echocardiogram showed a hypokinesis of the lateral heart wall. Echocardiography did not present any obstructions of the left ventricular outflow tract (LVOT). There were no heart valve defects or cardiac muscle hypertrophy found.

On the second day of ICU stay, the patient’s condition worsened. The patient was still sedated, GCS was 3–4 points, and pupils were narrow and reacted poorly. Due to cardiogenic shock, we abandoned the routine postoperative diagnostic methods (brain computer tomography and MRI). Hemodynamic parameters indicated severe cardiac injury and fluid requirements (cardiac output [CO] 2.4–3.0–2.68 L/min [norm 3–7], systemic vascular resistance 2573–1787–2680 dyn/s/cm^−5^ [norm 1100–1900], extravascular lung water index 6 mL/kg [norm 3–7], central venous pressure 4–10–5 mmHg [norm: 1–8]). The patient was still hypotensive (SBP 100–80 mmHg; DBP 40–30 mmHg, HR 98/min) with high lactate levels and unresponsive to the therapy that was used (IV fluids, vasopressors/catecholamines—NE, VP, DBT, ADR, continuous veno-venous hemodiafiltration). Severe coagulopathy developed within the first postoperative hours, and blood products and prothrombin complex were transfused. Despite the intensive treatment methods used, stabilization of the patient’s condition was not possible. In the second postoperative day, the patient developed multiple organ failure. The patient died in the ICU 51 h after the brain tumor resection. An autopsy is not a routine procedure in our country; therefore, it was not performed. Additionally, the family asked for no post-mortem examination.

## Discussion

Neurogenic stress cardiomyopathy (NSC), also called Takotsubo cardiomyopathy (TC) or apical ballooning syndrome, represents an acute heart failure syndrome with 4.1% hospital mortality and 30 days after mortality rate at the level of 5.9% [1]. It was found that in this syndrome (*n* = 1750) 46.8% of patients suffered from neurological and psychiatric disorders (9.4% patients with acute neurological disorder, 19.4% with chronic neurological disorders), whereas 15.3% of them had been diagnosed with coronary artery disease [2].

It can be observed after different acute neurological injuries (with or without intracranial hypertension) such as a traumatic brain injury, ischemic or a hemorrhagic stroke, especially a subarachnoid hemorrhage (SAH), central nervous system infections as well as after epileptic seizures [3]. In some studies (*n* = 32), physical stress and emotional stress were the trigger for TC in 59.1% and 40.9% of patients [2]. Differential diagnosis between NSC/TC and acute coronary syndrome should always be made [2].

Our case in the context of the patient’s illness history could not be considered as a potential candidate for such complications. Nevertheless, it was observed that NSC could occur in the perioperative period (also under anesthesia) because of stress sympathetic reflex stimulation, a surge in epinephrine levels, anaphylaxis and activation of so-called histamine-adrenergic cross talk [4]. NSC usually occurs in patients with SAH by post-bleed day (PBD) 2 [5]. By contrast, NSC was also diagnosed on PBD 7 in patients with hydrocephalus (in the absence of vasopressor therapy) [6]. The NSC, also known as a neurogenic stunned myocardium or broken heart syndrome, is also described as a reversible systolic dysfunction of the left ventricle (apical and mid-parts) with a balloon-like motion abnormality in the left ventricular wall [7]. In the study of 1750 patients, it became apparent that the most common type of TC was apical type (81.7% of patients), followed by mid-ventricular type (14.6%) and basal type (2.2%). The rarest type of TC (which was found in our patient) was focal type (1.5%) [1]. In such circumstances, one uses Mayo Clinic diagnostic criteria including: the presence of a transient abnormality in left ventricular wall motion, the presence of new electrocardiographic abnormalities or elevation in cardiac troponin levels and the absence of coronary artery disease, pheochromocytoma or myocarditis [1]. In some studies, NSC was defined as the presence of at least one marker of myocardial injury among the following markers: troponin ≥ 0.1, ejection fraction < 55%, long QT, T wave inversion and wall motion abnormalities [8]. NSC is expressed in EKG signs or in left ventricular wall motion abnormalities, the release of a myocardial necrosis enzyme and an increased BNP. In electrocardiography, differences with QT interval prolongation, ST segment depression, long QT syndrome, T-wave inversion and plenty of arrhythmias in NSC have been recognized [9, 10]. In the studies (*n* = 32), ST elevation was found in 53.1% of patients with TC whereas ST-depression/negative T with 43.8% [2]. To compare, in the other studies, TC with acute coronary syndrome (*n* = 1750), ST-segment elevation was found in 44.0% versus 51.2% (*p* = 0.003) and ST segment depression was found in 8.3% versus 31.1% (*p* < 0.001) patients [1]. EKG abnormalities occur in 25–75% of SAH patients while arrhythmias were recorded in all of them. Furthermore, ST elevation in 3% of the patients with intracranial tumors was also described [11]. According to the NSC definition, we observed very high troponin I and BNP levels in our patients and significant changes in EKG and transesophageal echocardiogram for cardiac injury as well as hemodynamic parameter abnormalities.

It is interesting in this case presentation that reports of a brain tumor as a source of NSC are lacking. Clinical manifestations of the above syndrome are tachycardia, ST-segment evolutions, myocardial stunning and circulatory impairment. Such biochemical markers as troponin 1 and BNP are elevated.

We observed very high glucose level at the beginning of cardiogenic shock but diabetes (major risk factor for acute coronary diseases) in our patient had never been diagnosed. High glucose level can be observed after steroids therapy or during endogenous adrenaline stress occurrence as well as adrenaline IV administration. Diabetes was considered as a risk factor only in 9.4% patients with TC [2].

The most popular theory of NSC is the disorders connected with the catecholamines system dysfunction that rapidly lead to a huge ejection of catecholamines. The acute brain injury rapidly provides the sympathetic hypertonus, overstimulation of the beta-1 and beta-2 receptors and simultaneously the reduction of the alpha-2 receptors activity. This process can lead to neurogenic stress cardiomyopathy, which could be seen as arrhythmias, reduced cerebral perfusion pressure or cariogenic shock and neuroinflammation. One of the potential etiologies for NSC is genetic [3].

Fluid load may even further depress circulation, causing pulmonary edema because of heart failure. We did not observe pulmonary edema in our patient in regards to clinical examinations as well as hemodynamic parameters measured. Nevertheless, we observed the lack of a hemodynamic reaction on the administration of catecholamines. Cardiac and vessel α, β receptors are blocked by a storm of endogenic catecholamines, so a response to catecholamines infusion is lacking.

Because of such mechanisms as cardiac insufficiency in NSC theoretically, levosimendan (calcium channel sensitizer) should also be considered especially if catecholamines inotropes are contraindicated [3]. Additionally, because epinephrine-mediated stunning is considered as a NSC factor, sympathomimetic drugs should be used with caution [4]. Neuroprotective effects of levosimendan in the prevention of inflammation and the normalization of the cerebral autoregulation as well as improvement of heart contractility without oxygen consumption increase are underlined [4]. Also, the beta-blockers in the period of sympathetic hyperactivity could be used in this disorder. Some studies have shown that beta-blocker use was associated with a less severe stroke [12]. Initial considerations on vasoactive administration in different types of cardiogenic shock are presented in American Heart Association Scientific Statement [13]. In patients with TC and cardiogenic shock due to LVOT obstruction therapeutic management including fluid resuscitation, cessation of inotropic therapy, IV beta-blocker and the use of intra-aortic balloon pump (IABP) resulted in non-inferiority survival as compared to patients without LVOT obstruction [2]. Our patient did not have LVOT obstruction, so with low cardiac output syndrome within cardiogenic shock, beta-blockers were contraindicated [2, 13]. Moreover, in the most recent European revascularization and non-ST-segment elevation ACS guidelines for a routine use in cardiogenic shock, application of IABP has been downgraded to Class AIII. Therefore, we did not consider that therapy [13].

We think that broader and more frequent recognition of the above complication should be achieved among doctors due to better knowledge of heart–brain connection especially in brain tumor resection.

Our case is unique because we have shown potentially lethal complications with sudden onset during a surgical procedure. The authors did not find published case reports indicating supratentorial brain tumor resection as a cause of NSC that started during the surgical procedure.

## Conclusions

1. Large intracranial tumor mass resection can be a cause of neurogenic stress cardiomyopathy.
2. More detailed perioperative cardiac evaluation and monitoring should be done in this group of patients
3. Catecholamines inotropes infusion was not effective, so calcium channel stimulators should be considered as a therapeutic option in the presented pathology.
